# Reduction of Skeletal Muscle Power in Adolescent Males Carrying H63D Mutation in the* HFE* Gene

**DOI:** 10.1155/2017/5313914

**Published:** 2017-12-06

**Authors:** Marcin Luszczyk, Barbara Kaczorowska-Hac, Ewa Milosz, Elzbieta Adamkiewicz-Drozynska, Ewa Ziemann, Radoslaw Laskowski, Damian Flis, Magdalena Rokicka-Hebel, Jedrzej Antosiewicz

**Affiliations:** ^1^Department of Physiology, Gdansk University of Physical Education and Sport, Kazimierza Gorskiego 1, 80-336 Gdansk, Poland; ^2^Department of Occupational Therapy, Gdansk University of Physical Education and Sport, Kazimierza Gorskiego 1, 80-336 Gdansk, Poland; ^3^Laboratory of Molecular Biology, Medical University of Gdansk, Marii Skłodowskiej-Curie 3A, 80-210 Gdansk, Poland; ^4^Department of Pediatrics, Hematology and Oncology, Medical University of Gdansk, Marii Skłodowskiej-Curie 3A, 80-210 Gdansk, Poland; ^5^Department of Physiology and Pharmacology, Gdansk University of Physical Education and Sport, Kazimierza Gorskiego 1, 80-210 Gdansk, Poland; ^6^Department of Bioenergetics and Nutrition, Gdansk University of Physical Education and Sport, Kazimierza Gorskiego 1, 80-336 Gdansk, Poland; ^7^Department of Tourism and Recreation, Gdansk University of Physical Education and Sport, Kazimierza Gorskiego 1, 80-336 Gdansk, Poland; ^8^Department of Bioenergetics and Physiology of Exercise, Medical University of Gdansk, Marii Skłodowskiej-Curie 3A, 80-210 Gdansk, Poland; ^9^Department of Biochemistry, Gdansk University of Physical Education and Sport, Kazimierza Gorskiego 1, 80-336 Gdansk, Poland

## Abstract

Iron overload resulting from the mutation of genes involved in iron metabolism or excess dietary intake has been reported to negatively influence human physical performance. The aim of this study was to test the hypothesis that adolescents bearing a hemochromatosis gene* (HFE)* mutation in contrast to adults with the same mutation will not experience iron accumulation and their aerobic capacity will be similar to that of age-matched controls. Thirteen boys participated in the study. Seven of them are carriers of H63D mutation in the* HFE* gene and six were wild type. Fitness levels were assessed using the cardiopulmonary exercise test. In addition, iron status and inflammatory markers were determined. We observed that cardiovascular fitness was significantly lower in the group bearing the* HFE* mutation compared to the control group. Moreover, the* HFE* mutation group achieved lower maximal power output compared to the control group. There were no differences in blood ferritin concentrations between the two groups which indicates similar amounts of stored iron. Obtained data do not confirm our hypothesis. On the contrary, it was demonstrated that* HFE* mutation is associated with a lower level of aerobic capacity, even in the absence of iron accumulation.

## 1. Introduction

Like oxygen, iron is essential for an organism functioning, although it in excess can be very toxic. The chemistry behind iron toxicity is relatively well documented; however, its molecular mechanism is still far from complete understanding.

The intracellular iron trafficking is organized in a way to keep the concentration of labile iron pool (LIP) low, to limit its ability to stimulate formation of the reactive oxygen species. An increase in cell iron drives ferritin biosynthesis, which leads to iron sequestration, lowering the LIP level. Consequently, cells with high iron stores are not necessarily characterized by high LIP levels [1]. Ferritin iron does not stimulate the reactive oxygen species (ROS) formation, thus being considered safe [2]. Nonetheless, studies on both humans and animals clearly demonstrate that an excessive iron accumulation, resulting from a medical condition (e.g., hemochromatosis) or dietary intake can have a range of deleterious effects including cancer, heart diseases, lower fitness level, and many others [3–5].

Hereditary hemochromatosis is an inherited disorder of iron overload characterized by an enhanced intestinal iron absorption. It is mutations in the hemochromatosis gene* (HFE)* that are the most often of hereditary hemochromatosis. Individuals homozygous for the mutation, which leads to C282Y substitution of tyrosine for cysteine at amino acid 282 in the* HFE* protein, are at increased risk of iron overload [6]. Hemochromatosis is a condition impairing synthesis of the master iron regulatory protein-hepcidin [7, 8]. Hepcidin downregulates ferroportin, known as the protein exporting iron out of cells in a gut and other cells [9]. Hence, low hepcidin levels are associated with an uncontrolled iron absorption. The most recent data show hemochromatosis patients to exhibit elevated serum ferritin, which was estimated to lead to an iron overload-related disease in 28% of 65-year-old males on average [10].

Individuals with a single copy of both C282Y and H63D (substitution of aspartic acid for histidine at amino acid 63) mutations in the* HFE* are also characterized by higher serum ferritin and transferrin saturation levels compared to people with neither of the* HFE* mutations; however, they are not at an enhanced risk of developing an iron overload-related disease [10]. Interestingly, even* HFE* heterozygotic genotypes might be associated with development or progression disease in the presence of concomitant diseases and possible environmental and epigenetic factors [11–13].

Furthermore, previous studies have demonstrated that iron overload may also negatively affect physical performance and muscle function in both human and animals. Hemochromatosis patients were found to have had lower peak oxygen uptake (VO_2peak_) [14], experiencing symptoms of weakness and fatigue [15]. Study included 40-year-old patients with iron stores 15–40 g and clinical manifestations often occurring [14, 15]. Some authors indicated that the H63D polymorphism, by resulting in hyperferritinemia, may have the potential to boost aerobic capacity in endurance athletes compared to sedentary population, despite the fact that other studies in adults men have not found a significant impact on VO_2peak_ and heart rate recovery from* HFE* mutations [16, 17].

Nevertheless there are no data concerning the effects of* HFE* mutation on physical performance in young people without any excessive iron accumulation or disease manifestation. In contrast to adults, clinical observations of young patients with* HFE* mutation have not revealed any symptoms pointing to diseases caused by iron overload. Thus, the purpose of this study was to evaluate if young boys bearing the* HFE* mutation (heterozygotes, having one copy of H63D) would differ in their physical performance from a boys presented with wild-type* HFE* gene, control group.

## 2. Material and Methods

### 2.1. Ethics Statement

This study was registered with the Medical University of Gdansk Clinical Trials Registry (ID: NKBBN/523/2013) and approved by the Independent Bioethics Commission for Research of Medical University of Gdansk according to the Helsinki Declaration. Before testing sessions, subjects and their parents received a verbal description of the experiment. Signed, written informed consent was obtained from all participants and their parents. Ethical approval was obtained for the referral of participants to their family physician upon detection of abnormal pathology results and review by the study medical officer.

### 2.2. Subjects

Thirteen unrelated, healthy adolescent boys originally volunteered to participate in this study. They were all students in local schools of Gdansk. The ethnic configuration of the group was 100% Caucasian. The subjects were examined towards mutation of* HFE* gene in a hospital hematology unit.

No attempt was made to recruit subjects who participated in competitive athletic training. The study was designed to examine adolescent males (mean age 17.0 ± 1.00 y). None of the subjects presented cardiac or respiratory problems such as postexercise asthma. The physical activity of all subjects include informal exercise (running, swimming, team games, or cycling) 2 hours per week in addition to their normal school physical education lessons (4 hours per week). Finally seven boys carriers of heterozygous HFE H63D mutation were formed experimental group and six boys with wild-type HFE gene, control group.

### 2.3. Experimental Design

Before enrollment in the study, the subjects underwent a complete physical check-up carried out by a physician, aimed at detecting a possible contraindication to the exercise test.

The subjects were measured and weighted using standard techniques. Body mass index was calculated as the body mass divided by the square of the body height. *Z*-score values were determined for body height, body mass, and body mass index [18]. Body surface area was calculated using the equation, which has been validated in infants, children, and adults [19]. Then the subjects were familiarized with the experimental procedure to reduce measurement error. Consecutively, the adolescents performed a cardiopulmonary exercise test (CPET). They were tested in a quiet room in the standard ambient conditions at least two hours after meals. The exercise test was preceded and followed by a cardiopulmonary function test and blood measurements.

### 2.4. Exercise Protocol

All participants performed a CPET in upright position on an electronically braked cycle ergometer (ViaSprint 150P; Ergoline, Bitz, Germany). After assessment at baseline cardiopulmonary values during a three-minute resting period the participants were asked to start cycling (1.50 Watts·kg^−1^) for five minutes (warm-up phase). Afterwards, the exercise intensity was increased by steps of 25 Watts·min^−1^ until exhaustion (test phase). Throughout the CPET, participants had to maintain a pedaling frequency between 50 and 60 revolutions·min^−1^. A test was considered to be at or near the maximal level when participants showed clinical signs of intense effort (e.g., unsteady biking, sweating, facial flushing, and clear unwillingness to continue exercising despite strong encouragement) and were unable to maintain the required pedaling speed and when at least one of the following criteria was met: a HR at peak exercise of >180 beats·min^−1^ or a respiratory exchange ratio at peak exercise of >1.00 [20].

*Measurements of Fitness. *During CPET, participants breathed through a facemask (Hans Rudolph, Kansas City, MO, USA) connected to a calibrated respiratory gas analysis system (Jaeger Oxycon Champion, Viasis Healthcare GmbH, Hochberg, Germany). Expired gas was passed through a flow meter, an oxygen analyzer, and a carbon dioxide analyzer. The flow meter and gas analyzers were connected to a computer, which calculated breath-by-breath minute ventilation, oxygen uptake, carbon dioxide production, and the respiratory exchange ratio averaged at ten-second intervals. Heart rate was monitored by telemetry (Polar Monitors, Electro, Kempele, Finland). All equipment was calibrated according to the instructions of the manufactured before exercise testing. Absolute values at peak exercise were calculated as the average value over the last 30 seconds prior to termination of the CPET as previously described [21]. Absolute oxygen uptake efficiency slope (OUES) was calculated as previously described [22]. The ventilator threshold was determined using the V-slope analysis method [23]. The relation between minute ventilation and carbon dioxide production was used to assess ventilatory efficiency during exercise [24] because among HH patients it has been linked to cardiac dysfunction [25]. Values of the relation between minute ventilation and carbon dioxide production < 30 are considered as a normal response to exercise [26, 27].

*Blood Sampling Protocols. *Subjects were tested for* HFE* mutations H63D. An early morning, fasting blood sample was collected from an antecubital vein for genetic testing for* HFE* mutations H63D which was performed using Real-Time Polymerase Chain Reaction. Two weeks later, subjects were admitted to exercise laboratory to perform the CPET. Before exercise and five minutes after exercise blood sample was collected from an antecubital vein into single-use containers with an EDTAK2 anticoagulant. After collection, all of the samples were immediately placed at 4°C and, within 10 min of collection, were centrifuged at 3000*g* at 4°C for 10 min. Iron metabolism was also assessed by measuring iron concentration, ferritin, and transferrin saturation (SYSMEX XE 2100, Architect ci 8200, and Test 1 SDL). None of the subjects trained during the day preceding the blood sampling. All preintervention and postintervention samples were analyzed in the same batch by technicians who were blinded to the group and order of the samples.

*Statistical Analysis. *Data are expressed as mean values ± standard error of the mean and were assessed using Student's unpaired *t*-test or Cochran-Cox test or Mann–Whitney test where appropriate. Results were considered significant at *p* ≤ 0.05.

## 3. Results

Subject characteristic is presented in Table 1. The mean age was comparable between the HFE group and control group (16.7 ± 0.41 were 17.2 ± 0.38 yr). Furthermore no significant differences in anthropometric parameters were found between both groups.

In order to confirm that young boys with* HFE* mutations were characterized by no iron accumulation, serum ferritin has been measured. The iron status parameters are presented in Table 2. Serum ferritin concentration was in normal physiological range and did not differ between the groups (*p* > 0.05). Moreover no differences were observed in serum iron and transferrin saturation. Ferritin is the best indicator of body iron stores in healthy persons, if it is not accompanied by inflammation [28, 29]. Thus, C-reactive protein has been measured as marker of inflammation and its values were in normal range in both groups and do not differ from each other (Table 2).

Exercise capacity measured by exercise time, peak oxygen uptake (VO_2peak_), and ventilator anaerobic threshold (VT) in* HFE* group were different compared with control subjects (Table 3).

VO_2peak_ measurement might be influenced by the patient's motivation and by the observer; thus relative oxygen uptake efficiency slope was used as a more objective method to study physical performance [22]. The OUES is derived from the relation between oxygen uptake and minute ventilation during incremental exercise [22]. All subjects reached a peak of respiratory exchange ratio ≥ 1.1 and were considered to perform maximal exercise.

**Figure 1:**
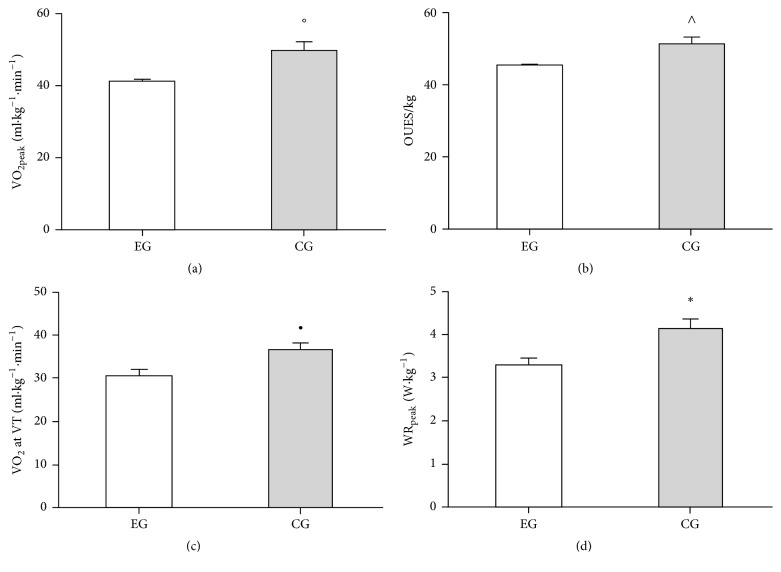
*Exercise performance*. Open and closed bars represent experimental (EG, *n* = 7) and control (CG, *n* = 6) groups, respectively. (a) Relative oxygen uptake at peak exercise; hemochromatosis group achieved less aerobic capacity (°*p* = 0.004). (b) Relative oxygen uptake efficiency slope; hemochromatosis group achieved less index of aerobic capacity (^∧^*p* = 0.020). (c) Relative oxygen uptake at the ventilator threshold; hemochromatosis group achieved less ventilatory threshold (^•^*p* = 0.018). (d) Relative work rate at peak exercise; hemochromatosis group performed less work rate (^*∗*^*p* = 0.027).

VO_2peak_ normalized for body mass was significantly lower in* HFE* group (Figure 1(a)). Value of respiratory exchange ratio was also comparable between the groups. OUES values (Figure 1(b)) determined from 100% exercise duration were significantly lower in subjects bearing* HFE* mutation. VT which is another indicator of aerobic performance was also determined.

We observed that value of oxygen uptake at the VT was significantly lower in* HFE* group compared to the control (Figure 1(c)). Relative peak power output elicited during the exhaustion test showed much higher values in control group compared to* HFE* (Figure 1(d)).

Furthermore max minute ventilation was significantly lower in* HFE* group compared to control subjects (Table 3).

All together data indicates that young boys bearing* HFE* mutations demonstrated lower aerobic performance and lower aerobic power then their contemporary age match, whereas ventilator efficiency described by relation between minute ventilation and carbon dioxide production was comparable between the groups (Table 3).

## 4. Discussion

The H63D mutation of* HFE* gene is cosmopolitan but greater in white populations (10% to 29%) of western Europe [6], whereas H63D simple heterozygosity is more prevalent (23.6% to 31.1%) in populations of northern European and our population [30, 31]. Despite these high prevalence and association with the risk of iron overload the population of* HFE* H63D heterozygotes has not been widely examined, especially in children. Furthermore the intensity of iron storage and influence on physical performance in* HFE* carriers in the developmental age are not well elucidated. Notably, since iron accumulation is a prolonged process, iron overload is rarely observed in children [30]. Thus early diagnosis, monitoring, and treatment are essential.

In the present study for the first time we demonstrate that adolescent boys in contrast to adults patients, bearing the H63D* HFE* gene mutation, are characterized by lower cardiorespiratory fitness. In the previously published studies performed on adult hemochromatosis patients, the lower cardiorespiratory fitness and disturbance in iron metabolism were observed [14]. In this study, we hypothesized that adolescent boys, heterozygotes for* HFE* gene, and lacking any iron overload would exhibit a physical performance matching the control group in their age. These assumption has not been confirmed.

There are some direct and indirect evidences that excess iron accumulation might impair muscle function and lower physical performance. Iron overload may be induced by diet and number of mutations in genes encoding proteins of iron metabolism, one of which is* HFE*. Studies on animals demonstrated that intraperitoneal injections of iron lead to iron overload. Moreover, muscle iron accumulation was associated with performed lower work on an endurance test and less force in a strength test [32].

Data obtained on individuals with a hereditary hemochromatosis supported the animal studies. This study clearly demonstrated that genetic haemochromatosis is associated with lower exercise capacity [14]. It is known that, in excess, iron accumulation leads to an iron-dependent ROS formation, inducing an oxidative stress, which impairs force generation during exercise [33, 34]. It is a puzzling phenomenon since theoretically stored iron is located in ferritin, unable to stimulate the ROS formation. It is only the labile iron pool, iron loosely bound to low molecular weight compounds like nucleotides amino acids and so forth, that can drive the formation of ROS [2]. Cells exposed to increasing level of LIP respond with adaptive changes, one of which is the rise in ferritin biosynthesis [35]. Recently, it has been shown that c-Jun n-terminal kinase, which belongs to stress-activated protein kinases, stimulates ferritin degradation and iron-dependent ROS formation [36, 37]. Consequently, it can be assumed that during stress condition stored iron may be partially liberated, giving rise to the iron-dependent ROS formation [36, 38]. The c-Jun n-terminal kinase activation is regulated by different signaling pathways, for example, heat shock protein 70 block c-Jun n-terminal kinase activation and one of the factors which could stimulate the rise of heat shock protein 70 that is exercise [39]. Therefore, it can be expected that liberation of iron from ferritin is determined by an organism's response to stress as well as the amount of iron stored. In that way it can determine the iron toxicity.

The available data clearly demonstrate that the amount of stored iron, resulting from cardiac dysfunction, might influence human performance. Study conducted on young men (20–49 y) revealed that higher blood ferritin correlates negatively with cardiorespiratory fitness, even if the ferritin concentration was in reference range (>100 < 150 ng·ml^−1^) [40]. In the current study we observed that the* HFE* mutants are characterized by the lower power output generated at VO_2peak_, as well as lower max minute ventilation compared to control subjects.

This may be the case due to the first group having weaker respiratory muscle. On average the subjects in our study were 16.7 years old, which corresponds with the age at which some liver iron accumulation was suggested to may have already occurred [15]. It seems that assessment of aerobic fitness capacity may be useful tool in diagnosis of H63D mutation before typical sights of onset of disease. Still, for the purpose of the study, no data on the skeletal muscle iron concentration was available.

Serum ferritin concentration (most often used as a marker of stored iron) did not differ between the* HFE* mutants and control subjects, with a mean concentration of 46 ng·ml^−1^.

These data suggest that iron accumulation is rather not responsible for the lower fitness level observed in* HFE* mutants. Studies performed on hemochromatosis patients somewhat support this conclusion. It was demonstrated that even when treated with a regular phlebotomy (the best way to lower body iron stores) hemochromatosis patients did not improve their exercise capacity [14]. Still, considering that these observations were made for 50-year-old patients, it is possible that some iron-related damage had happened before phlebotomy was started.

Another way to explain lower fitness level of the boys bearing the* HFE* gene mutation is iron accumulation in skeletal muscle, potentially not reflected in the serum ferritin level.

It may also be the case that the mutation affects organism function in ways that still remain to be recognized.

## 5. Conclusion

In conclusion, our data clearly demonstrates that young boys, bearing the* HFE* gene mutation, exhibit an impaired cardiorespiratory function and lower skeletal muscle force compared to wild-type carriers. Still, these differences cannot be explained by implications of an excessive iron accumulation. Iron metabolism in hemochromatosis patients requires thus further investigation to address the following limitations of our study: a small number of subjects and lack of assessment of anaerobic power in participants.

## Figures and Tables

**Table 1:** Anthropometrics characteristics of the subjects.

Variables	EG	CG	*p* value
	(*n* = 7)	(*n* = 6)	
Age (y)	16.7 ± 0.41	17.2 ± 0.38	0.400
Height (cm)	176.6 ± 3.07	175.0 ± 2.84	0.718
Height (*z*-score)	−0.10 ± 0.51	−0.43 ± 0.46	0.651
Weight (kg)	67.2 ± 4.84	62.3 ± 3.68	0.454
Weight (*z*-score)	−0.08 ± 0.47	−0.60 ± 0.34	0.415
BMI (kg·m^−2^)	21.4 ± 0.79	20.3 ± 0.89	0.391
BMI (*z*-score)	0.05 ± 0.31	−0.42 ± 0.29	0.296
BSA (m^2^)	1.81 ± 0.08	1.73 ± 0.06	0.486

Results are shown as means ± SEM. No statistically significant differences were noted between the groups in any of these measures. EG, hemochromatosis group; CG, control group; BMI, body mass index; BSA, body surface area.

**Table 2:** Iron status parameters in control and *HFE* mutation group.

Variables	EG	CG	*p* value
	(*n* = 7)	(*n* = 6)	
Fe (ug·dl^−1^)	128.3 ± 20.7	85.7 ± 11.2	0.114
Ferritin (ng·dl^−1^)	43.6 ± 7.94	45.1 ± 8.20	0.902
Ts (%)	35.6 ± 4.62	25.3 ± 3.07	0.103
CRP (mg·dl^−1^)	0.79 ± 0.26	0.66 ± 0.50	0.350

Results are shown as means ± SEM. No statistically significant differences were noted between the groups in any of these measures. EG, hemochromatosis group; CG, control group; Fe, iron; Ts, transferrin saturation; CRP, C- reactive protein.

**Table 3:** Cardiopulmonary exercise test parameters in control and *HFE* mutation group.

Variables	EG	CG	*p* value
	(*n* = 7)	(*n* = 6)	
Exercise time (s)	327 ± 34	345 ± 14	0.657
HR_peak_ (beats·min^−1^)	190 ± 4	191 ± 4	0.871
WR_peak_ (W)	220.4 ± 16.8	254.1 ± 7.98	0.053
METs_peak_	11.7 ± 0.31	14.1 ± 0.68	0.006^*∗*^
VO_2peak_ (L·min^−1^)	2.73 ± 0.14	3.08 ± 0.16	0.133
VT (% VO_2peak_)	74.1 ± 2.31	73.4 ± 1.31	0.793
VE_peak_ (L·min^−1^)	88.9 ± 4.34	112.6 ± 6.24	0.008^*∗*^
VE/VCO_2slope_	24.4 ± 1.15	23.8 ± 0.79	0.687
RER_peak_	1.24 ± 0.04	1.25 ± 0.02	0.756

Results are shown as means ± SEM. ^*∗*^Statistical significant difference; EG, hemochromatosis group; CG, control group; HR_peak_, heart rate at peak exercise; WR_peak_, work rate at peak exercise; METs, metabolic equivalent; VO_2peak_, oxygen uptake at peak exercise; VT, ventilatory threshold; VE_peak_, minute ventilation at peak exercise; VE/VCO_2slope_ relation between minute ventilation and carbon dioxide production; RER_peak_, respiratory exchange ratio.

## References

[1] Epsztejn S., Glickstein H., Picard V. (1999). H-ferritin subunit overexpression in erythroid cells reduces the oxidative stress response and induces multidrug resistance properties. *Blood*.

[2] Kruszewski M. (2003). Labile iron pool: the main determinant of cellular response to oxidative stress. *Mutation Research - Fundamental and Molecular Mechanisms of Mutagenesis*.

[3] Bonilla S. F., Melin-Aldana H., Whitington P. F. (2010). Relationship of proximal renal tubular dysgenesis and fetal liver injury in neonatal hemochromatosis. *Pediatric Research*.

[4] MacDonald H. B., Salonen J. T., Nyyssonen K. (1993). High stored iron levels are associated with excess risk of myocardial infarction in Eastern Finnish men. *Circulation*.

[5] Chicharro J. L., Hoyos J., Gómez-Gallego F. (2004). Mutations in the hereditary haemochromatosis gene HFE in professional endurance athletes. *British Journal of Sports Medicine*.

[6] Barton J. C., Edwards C. Q., Acton R. T. (2015). *HFE* gene: structure, function, mutations, and associated iron abnormalities. *Gene*.

[7] Alexander J., Kowdley K. V. (2009). HFE-associated hereditary hemochromatosis. *Genetics in Medicine*.

[8] Gurrin L. C., Osborne N. J., Constantine C. C. (2008). The Natural History of Serum Iron Indices for HFE C282Y Homozygosity Associated With Hereditary Hemochromatosis. *Gastroenterology*.

[9] De Domenico I., Ward D. M., Langelier C. (2007). The molecular mechanism of hepcidin-mediated ferroportin down-regulation. *Molecular Biology of the Cell (MBoC)*.

[10] Gurrin L. C., Bertalli N. A., Dalton G. W. (2009). HFE C282Y/H63D compound heterozygotes are at low risk of hemochromatosis-related morbidity. *Hepatology*.

[11] Adams P. C., Walker A. P., Acton R. T. (2001). A primer for predicting risk of disease in HFE-linked hemochromatosis. *Genetic Testing*.

[12] Beutler E., Felitti V. J., Koziol J. A., Ho N. J., Gelbart T. (2002). Penetrance of 845G → A (C282Y) *HFE* hereditary haemochromatosis mutation in the USA. *The Lancet*.

[13] Islek A., Inci A., Sayar E., Yilmaz A., Uzun O. C., Artan R. (2016). HFE-related hereditary hemochromatosis is not invariably a disease of adulthood: Importance of early diagnosis and phlebotomy in childhood. *Journal of Pediatric Gastroenterology and Nutrition*.

[14] Davidsen E. S., Liseth K., Omvik P., Hervig T., Gerdts E. (2007). Reduced exercise capacity in genetic haemochromatosis. *European Journal of Preventive Cardiology*.

[15] Brandhagen D. J., Fairbanks V. F., Baldus W. (2002). Recognition and management of hereditary hemochromatosis. *American Family Physician*.

[16] Grealy R., Herruer J., Smith C. L. E., Hiller D., Haseler L. J., Griffiths L. R. (2015). Evaluation of a 7-gene genetic profile for athletic endurance phenotype in ironman championship triathletes. *PLoS ONE*.

[17] Arena R., Shizukuda Y., Bolan C. D. (2007). Heart rate recovery is lower following supine exercise in asymptomatic hereditary hemochromatosis subjects compared with healthy controls. *Journal of Cardiopulmonary Rehabilitation and Prevention*.

[18] Kułaga Z., Rożdżyńska A., Palczewska I. (2010). Percentile charts of height, body mass and body mass index in children and adolescents in Poland – results of the OLAF study. *Standardy Medyczne/Pediatria*.

[19] Haycock G. B., Schwartz G. J., Wisotsky D. H. (1978). Geometric method for measuring body surface area: A height-weight formula validated in infants, children, and adults. *Journal of Pediatrics*.

[20] Barker A. R., Williams C. A., Jones A. M., Armstrong N. (2011). Establishing maximal oxygen uptake in young people during a ramp cycle test to exhaustion. *British Journal of Sports Medicine*.

[21] Bongers B. C., Hulzebos E. H. J., van Brussel M., Takken T. (2012). *Pediatric norms for cardiopulmonary exercise testing in relation to gender and age*.

[22] Baba R., Nagashima M., Goto M. (1996). Oxygen uptake efficiency slope: a new index of cardiorespiratory functional reserve derived from the relation between oxygen uptake and minute ventilation during incremental exercise. *Journal of the American College of Cardiology*.

[23] Beaver W. L., Wasserman K., Whipp B. J. (1986). A new method for detecting anaerobic threshold by gas exchange. *Journal of Applied Physiology*.

[24] Paridon S. M., Alpert B. S., Boas S. R. (2006). Clinical stress testing in the pediatric age group: A statement from the American Heart Association council on cardiovascular disease in the young, committee on atherosclerosis, hypertension, and obesity in youth. *Circulation*.

[25] Shizukuda Y., Bolan C. D., Tripodi D. J. (2006). Left Ventricular Systolic Function During Stress Echocardiography Exercise in Subjects With Asymptomatic Hereditary Hemochromatosis. *American Journal of Cardiology*.

[26] Shizukuda Y., Smith K. P., Tripodi D. J. (2012). Changes in exercise capacity in subjects with cardiac asymptomatic hereditary hemochromatosis during a follow-up after 5 yrs. *American Journal of Physical Medicine & Rehabilitation*.

[27] Witte D. L., Crosby W. H., Edwards C. Q., Fairbanks V. F., Mitros F. A. (1996). Hereditary hemochromatosis. *Clinica Chimica Acta*.

[28] Bell J. D., Kincaid W. R., Morgan R. G. (1980). Serum ferritin assay and bone-marrow iron stores in patients on maintenance hemodialysis. *Kidney International*.

[29] Brown R. D., Benfatto J., Gibson J., Kronenberg H. (1988). Red cell ferritin and iron stores in patients with chronic disease. *European Journal of Haematology*.

[30] Barbara K.-H., Marcin L., Jedrzej A. (2016). The impact of H63D HFE gene carriage on hemoglobin and iron status in children. *Annals of Hematology*.

[31] Kaczorowska-Hac B., Luszczyk M., Antosiewicz J. (2017). HFE Gene Mutations and Iron Status in 100 Healthy Polish Children. *Journal of Pediatric Hematology/Oncology*.

[32] Reardon T. F., Allen D. G. (2009). Iron injections in mice increase skeletal muscle iron content, induce oxidative stress and reduce exercise performance. *Experimental Physiology*.

[33] Lawler J. M., Cline C. C., Hu Z., Coast J. R. (1997). Effect of oxidative stress and acidosis on diaphragm contractile function. *American Journal of Physiology-Regulatory, Integrative and Comparative Physiology*.

[34] Reid M. B., Stokić D. S., Koch S. M., Khawli F. A., Leis A. A. (1994). N-acetylcysteine inhibits muscle fatigue in humans. *The Journal of Clinical Investigation*.

[35] Chevion M., Leibowitz S., Aye N. N. (2008). Heart protection by ischemic preconditioning: a novel pathway initiated by iron and mediated by ferritin. *Journal of Molecular and Cellular Cardiology*.

[36] Antosiewicz J., Ziolkowski W., Kaczor J. J., Herman-Antosiewicz A. (2007). Tumor necrosis factor-*α*-induced reactive oxygen species formation is mediated by JNK1-dependent ferritin degradation and elevation of labile iron pool. *Free Radical Biology & Medicine*.

[37] Borkowska A., Sielicka-Dudzin A., Herman-Antosiewicz A. (2012). Diallyl trisulfide-induced prostate cancer cell death is associated with Akt/PKB dephosphorylation mediated by P-p66shc. *European Journal of Nutrition*.

[38] Borkowska A., Sielicka-Dudzin A., Herman-Antosiewicz A., Halon M., Wozniak M., Antosiewicz J. (2011). P66Shc mediated ferritin degradation-A novel mechanism of ROS formation. *Free Radical Biology & Medicine*.

[39] Thompson H. S., Maynard E. B., Morales E. R., Scordilis S. P. (2003). Exercise-induced HSP27, HSP70 and MAPK responses in human skeletal muscle. *Acta Physiologica Scandinavica*.

[40] Mainous A. G., Díaz V. A. (2009). Relation of serum ferritin level to cardiovascular fitness among young men. *American Journal of Cardiology*.

